# Identifying Accessibility and Equity Defects of Elderly Care Services in Developing Countries: Insights From Xiamen City

**DOI:** 10.1155/2024/9340456

**Published:** 2024-09-23

**Authors:** Linjiang Wei, Ya Fang, Liangwen Zhang

**Affiliations:** ^1^ State Key Laboratory of Molecular Vaccinology and Molecular Diagnostics School of Public Health Xiamen University, Xiamen, China; ^2^ Key Laboratory of Health Technology Assessment of Fujian Province School of Public Health Xiamen University, Xiamen, China; ^3^ State Key Laboratory of Vaccines for Infectious Diseases Xiang An Biomedicine Laboratory Xiamen University, Xiamen, China

**Keywords:** elderly care service facilities, health equity, nursing, older adults, spatial accessibility

## Abstract

**Background:** The global aging population has raised concerns about the fair distribution of elderly care resources. China, as the largest developing country, has made efforts to address population aging challenges. However, equitable distribution of elderly care resources remains a concern. This study analyzed the spatial layout and accessibility of elderly care service facilities (ECSFs) in Xiamen City to improve resource allocation and enhance the elderly care system in China. The findings provide valuable insights for other developing countries and regions seeking to improve their own elderly care resource allocation.

**Methods:** Xiamen City was chosen as the research area due to its unique geographical location and advanced information technology infrastructure. The study examined the spatial distribution, accessibility, and equity of ECSFs. Using Python, point of interest (POI) data were collected and evaluated using the kernel density method and two-step floating catchment area method. Spatial autocorrelation analysis identified areas of aggregation and dispersion in ECSF accessibility.

**Results:** Xiamen currently has 660 ECSFs, including nursing homes, adult day care centers, home care agencies, and rural elderly care homes. The analysis revealed spatial disparities, with ECSFs clustering primarily in the central area of Xiamen Island. Significant differences in accessibility were found among the four types of ECSFs. Spatial autocorrelation analysis identified cold and hot spot areas, indicating variations in accessibility across regions.

**Conclusion:** Xiamen City has made progress in allocating elderly care resources and constructing service facilities. However, equity in resource allocation remains a concern. Areas with limited accessibility were identified, leading to unequal access to elderly care resources and timely physical care. To address these challenges, decision-making departments should consider increasing facilities, improving transportation, enhancing macro planning, and improving facility service quality and accessibility. These measures will optimize ECSF accessibility and promote equitable distribution of elderly care resources.

## 1. Introduction

In recent years, the global aging process has continued to deepen [1], especially after the outbreak of the COVID-19 pandemic, where the elderly population became a key focus of care due to their weakened immune systems [2]. As the country with the largest aging population in the developing world, the demand for medical care, rehabilitation, and elderly care resources for the elderly in China continues to increase [3, 4]. Informal care is facing challenges such as inadequate care manpower and low care quality, while formal care has begun to receive special attention. Under tremendous pressure, the government has made many attempts and introduced a series of positive policies [5, 6], which has led to the golden period of elderly care service system construction [7]. The elderly care industry has flourished, the types and numbers of elderly care service facilities (ECSFs) have started to surge, and market-oriented elderly care methods such as home care, institutional elderly care, and community elderly care have emerged one after another. However, it is worth noting that some studies have shown that there are still problems with the equity of elderly care resource allocation in China [8]. There is an obvious supply–demand mismatch, and even some existing facilities in developed regions do not meet the fair and reasonable planning requirements [9–12].

Due to the decline in physical functioning, the elderly population has reduced adaptive capacity to the environment and is more susceptible to the influence of external environmental factors [13–16]. Therefore, for elderly individuals living in areas with fair allocation of elderly resources and reasonable spatial distribution of ECSFs, it is easier for them to access the necessary elderly resources and receive timely physical care at ECSFs. This enables them to meet their basic life needs at a lower transportation cost, ensuring their physical and mental well-being and enhancing their quality of life [17].

Accessibility assessment is a key indicator for measuring the equity and rationality of spatial distribution [18–20], and it has been widely applied in evaluating the layout of public service facilities such as education, healthcare, parks, and green spaces [20]. It can reflect the ease or difficulty for the elderly population in accessing elderly care services.

Currently, there are many methods used by scholars for accessibility assessment in the field of elderly resource allocation, and the corresponding indicators are also diverse [21]. However, they mainly involve two analytical approaches: accessibility assessment based on opportunity accumulation and accessibility assessment based on spatial interaction. Accessibility assessment based on opportunity accumulation focuses on quantifying the amount of resources that facility points or residential points can access within a certain time threshold. Spatial interaction models are geographic methods derived from Newton's universal law of gravitation. Their advantage lies in their ability to utilize relevant data to elucidate the interaction between supply and demand of elderly resources and to analyze the impact of geographical accessibility on individual behavior while evaluating individual spatial accessibility. In summary, the accessibility assessment methods based on opportunity accumulation consider multiple factors such as transportation networks, distribution of service facilities, and population density. They delve deeper into the cumulative effects of resources and the consideration of temporal factors. However, the application of such spatial analysis methods in the field of elderly resource allocation is still relatively limited.

From the perspective of research content, there are still some limitations in the current studies on accessibility assessment of elderly resources. First, the research scale is not sufficiently refined. Existing studies often treat a city or a specific urban area as a whole, without delving into the specific accessibility of each residential cluster. This overlooks individual travel time and living range, which may lead to research conclusions that do not align with reality [22]. Second, the research areas mainly focus on the core urban areas or specific regions of a city, with fewer comprehensive assessments of accessibility across the entire city, neglecting to address the disparities between rural and urban areas. Furthermore, there are various types of ECSFs, but most studies do not classify them or only focus on the accessibility of a specific type of facility [23]. Lastly, existing research data often come from government official statistical yearbooks, which may lack detailed information and may not be promptly updated [24].

**Figure 1:**
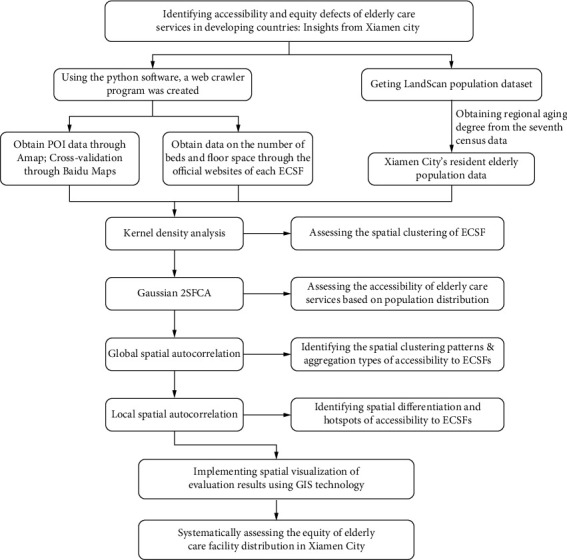
Research framework.

In summary, while the elderly care industry in China has been rapidly developing, it has also revealed issues such as unfair resource allocation and mismatched supply and demand. Faced with the dilemma of elderly individuals being unable to access care services equitably, there are currently some deficiencies in the methods and content of evaluating the accessibility of elderly care resources. Therefore, this study establishes the following research logic and objectives: First, by employing Python programming language, a web crawler program was developed to obtain the latest point of interest (POI) data on ECSFs in Xiamen City. These data include information such as name, category, coordinates, and classification. Second, using kernel density estimation, two-step floating catchment area (2SFCA) method, and spatial autocorrelation analysis, a comprehensive evaluation was conducted on the spatial accessibility and equity of various types of ECSFs in Xiamen, including nursing homes (NHs), adult day care centers (ADCs), rural health clinics (RHCs), and home care agencies (HCAs) (see Figure 1 for details).

The aim is to identify potential issues in the allocation of elderly care resources in Xiamen, expand the theoretical knowledge to address resource allocation problems, provide a basis for scientifically formulating resource allocation plans, rationalize the layout of elderly care service planning, enhance the quality of care for the elderly population, and improve the overall elderly care service system. Moreover, this study aims to provide valuable insights that can be applied to other cities in China as well as various developing countries and regions.

## 2. Methods

### 2.1. Research Site

**Figure 2:**
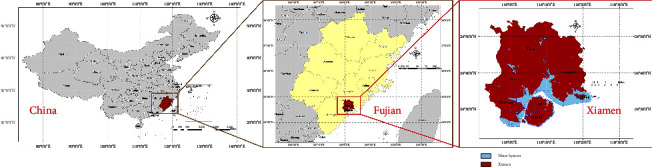
Research site.

**Figure 3:**
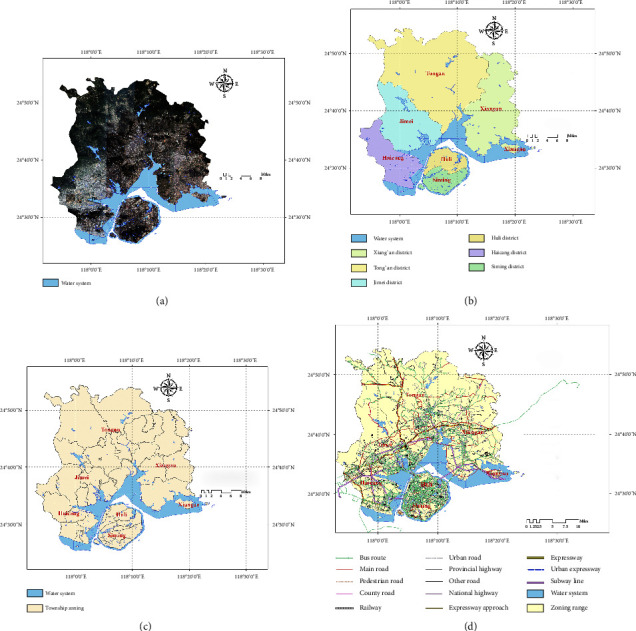
(a) The Xiamen satellite map, (b) the extent of administrative divisions in Xiamen, (c) the extent of street and township divisions in Xiamen, and (d) road traffic in Xiamen.

Xiamen is located in the southeast of Fujian Province in East China (the specific location of Xiamen City can be seen in Figure 2, and the satellite image is shown in Figure 3(a)). As of 2021, Xiamen has six districts, including Siming, Huli, Jimei, Haicang, Tong'an, and Xiang'an, with 37 streets and eight towns (as shown in Figures 3(b) and 3(c)). The total area is 1700.61 square kilometers, with a permanent population of 5.28 million [25]. The total length of roads in the city (excluding natural village roads) is 2223.13 km, including 259.17 km of national roads, 240.4 km of provincial roads, 394.9 km of county roads, 657.4 km of township roads, and 671.27 km of village roads (as shown in Figure 3(d)).

As a developed city on the southeastern coast of China, Xiamen has a high level of economic development, a strong foundation for elderly care services, well-developed information technology infrastructure, transparent access to various types of information, and easy accessibility. The city has set goals for future elderly care service system development: By 2025, a batch of urban convenient living circles with complete functions, smart and convenient services, and high-quality service and living harmony will be built, in accordance with the requirements of a “15-min living circle,” to reduce the difficulty in obtaining elderly care services. At the same time, the city continues to focus on the equity of elderly care service access and aims to establish a sound elderly care service system based on home care, supported by the community, supplemented by institutions, and integrating medical and elderly care by 2035. This will expand elderly care services from specific populations to the general public, transform social welfare from “deficiency compensation” to “universal coverage,” and ultimately achieve a minimum of 40 senior care beds per thousand elderly people and a per capita land area for ECSFs of no less than 0.1 square meters in urban and rural areas [26]. As the global aging population increases, economic development and national economic strength continue to improve in China and other developing countries, and more and more cities will follow a development trajectory similar to Xiamen. Therefore, Xiamen City was selected as the research area for this study to clarify the equity of ECSF construction in developed Chinese cities and to provide experience and reference for other developing countries and regions.

### 2.2. Data Acquisition

According to the 15-min living circle construction criteria and the spatial layout planning of ECSFs in Xiamen City, this study classified the ECSFs into four categories: A (NHs that provide long-term centralized nursing services, with a service radius of 10–15 km), B (ADCs that provide day care services, with a service radius of 1–1.5 km), C (REHs that mainly serve rural residents, providing comprehensive elderly care services based on home care and community support, with a service radius of about 1 km), and D (HCAs that mainly serve urban residents, providing home-based services within 1 km). The specific service radius was determined based on the number of beds in each facility.

To conduct a scientific evaluation of the layout of ECSFs, it is necessary to analyze and study them in the context of geographic space. Traditional methods of data acquisition often rely on sources such as statistical yearbooks and registration data from civil affairs bureaus, utilizing methods such as surveys and door-to-door inquiries. However, these approaches are time-consuming and labor-intensive and may face challenges in obtaining data due to issues such as permissions. While secondary statistics from government or health departments may have higher reliability, they often lack detailed information on individual medical institutions due to access restrictions. Moreover, official secondary data are typically updated only once a year or even less frequently, leading to potential data lag. In the current context of various medical vertical portals and Internet mapping service providers, Internet data offer advantages such as large volume, timeliness, and low acquisition costs. Therefore, this study starts with Internet data and utilizes Python web scraping techniques and Internet open platforms to collect data related to elderly care facilities and the associated assessments.

Among the collected data, the information on elderly care facilities and road traffic POIs was collected in March 2023. The data on population aggregation points were collected at the end of 2021, and the estimation of the elderly population in each administrative region was based on the results of the seventh national population census of China.

The POI web crawler software used in this study was developed using Python 3.10. The road traffic data were sourced from Amap (https://ditu.amap.com/) and Baidu Maps (https://map.baidu.com/). The data on elderly care institutions were collected from the Xiamen Municipal Government's Convenience Platform (https://www.ixiamen.org.cn/) and various official websites of the care institutions, such as Xiangyu nursing home (https://xiangyuciai.com/) and HA nursing home (https://www.hanursing.com/). Python 3.10 was employed for statistical analysis, while ArcGIS 8.0 software was utilized for creating the statistical maps.

#### 2.2.1. ECSF

This study utilizes the Amap Developer Platform and its Web development service module to search for POIs by developing a Python web crawler script. The script is designed to match the specified data request format in order to obtain POI data for elderly care institutions with geospatial information, including names, categories, coordinates, and classifications. To validate the results, cross-validation is performed using POI data obtained from Baidu Maps.

After the statistical classification of POI data, the number of beds and floor area data were obtained by querying the official websites of various ECSFs.  Table 1 shows an example of obtaining data.

#### 2.2.2. Demographic

In existing research on the quantification of accessibility and equity of ECSFs, population data from regional statistical yearbooks are often used as the demand-side data. The statistical scale is mostly at the level of administrative regions or streets, which can lead to homogenization of the calculation results. To obtain more realistic results in accessibility calculations, this study narrowed the statistical scale and used population data within a 1 km ∗ 1 km range as the demand scale for accessibility evaluation.

The population spatial distribution grid data of Xiamen City are sourced from the 2021 LandScan population dataset by Oak Ridge National Laboratory (https://landscan.ornl.gov/). These data provide community-level population distribution data worldwide, with a spatial resolution of 1 km × 1 km, sourced from the total amount of environmental data over a period of time. In this study, we used these data in combination with the elderly proportion in each administrative region in the seventh national census report (see Table 2) to estimate the number of elderly people in the study area.

### 2.3. Statistical Methods

In this study, a series of spatial statistical methods were employed to assess the fairness of elderly care facility distribution in Xiamen City. Specifically, we utilized the following methods: First, kernel density analysis was used to illustrate the spatial clustering patterns of different types of elderly care facilities. However, this method only displays the density quantity of facilities in space and does not reflect the actual relationship between the elderly population and the availability of care resources. Therefore, in this study, we employed the Gaussian 2SFCA (Ga2SFCA) method to delve deeper into the accessibility of various types of care institutions. Moreover, we conducted global spatial autocorrelation analysis and local spatial autocorrelation analysis to identify the spatial heterogeneity of elderly care facility accessibility in Xiamen City and pinpoint specific cold and hot spot areas. This comprehensive evaluation allowed this study to provide a more thorough assessment of the elderly care facilities in Xiamen. Here is a brief introduction to the specific statistical methods employed.

#### 2.3.1. Kernel Density Analysis

Kernel density estimation is a nonparametric method used for spatial analysis of point elements. It estimates the density variation of point distributions using a moving unit and reveals the clustering patterns of point elements on a spatial scale, thus reflecting the accessibility of elderly resources [27]. The formula for calculating kernel density is given by the following equation:

(1) $$\begin{array}{c} \lambda_{s}=\overset{n}{\underset{l=1}{\sum }}\frac{1}{\pi r^{2}}\varphi \left(\frac{d_{ls}}{r}\right), \end{array}$$

where *λ*_*s*_ represents the kernel density value at grid *s*, *r* denotes the search radius, *n* indicates the total number of POI points, *d*_*ls*_ represents the distance between POI points, and *φ* represents the weight.

#### 2.3.2. Ga2SFCA

The 2SFCA method, as a mainstream accessibility evaluation method, has received widespread attention from scholars and has been introduced into research on elderly care resource allocation [28]; it can comprehensively consider the capacity, distribution, type, distance, and travel mode of service facilities and is easy to be personalized designed.

The Ga2SFCA was proposed by Dai in 2010. He used a Gaussian function as the distance decay function *g*(*d*_*ij*_) [29] within the 2SFCA search domain for the accessibility evaluation of medical facilities. The specific calculation method can be found in the following equation:

(2) $$\begin{array}{c} A_{i}=\overset{n}{\underset{j=1}{\sum }}\frac{S_{j}f\left(d_{ij}\right)}{\sum_{k=1}^{m}D_{k}f\left(d_{kj}\right)}, \end{array}$$

where *A*_*i*_ represents the accessibility of demand point *I* to obtain pension services, *d*_*ij*_ represents the road network distance between supply point *j* and demand point *I*, *d*_*kj*_ represents the distance between supply point *j* and demand point *k*, and *Df*(*d*_*ij*_) represents the distance decay function. It can also be further expressed as the following equation:

(3) $$\begin{array}{c} f\left(d_{ij}\right)=\left\{\begin{array}{cc} g\left(d_{ij}\right), & d_{ij}\leq d_{0}\\ 0, & d_{ij}>d_{0} \end{array}\right\}, \end{array}$$

where *f*(*d*_*ij*_) represents the distance decay function, *d*_*ij*_ represents the distance between the supply point *j* and the demand point *i*, *d*_0_ represents the search range, and *g*(*d*_*ij*_) represents the distance decay function within the search range *d*_0_. *g*(*d*_*ij*_) is introduced as a modified calculation of the original 2SFCA method by introducing the decay function. In the original 2SFCA method, *g*(*d*_*ij*_) is always a constant value of 1.

The proposed method differs from the original 2SFCA method in several aspects. First, this method enables a random exploration of the entire search space to seek the global optimum. In addition, it employs an adjustment of the search step length and direction, along with a sampling strategy using Gaussian distribution, to perform a more refined search in the neighborhood, aiming to find solutions that are closer to the local optimum. Second, it effectively deals with uncertainties and noise within the search space by employing an adaptive adjustment of the search step length and direction, as well as a sampling strategy using Gaussian distribution. This robustness enhances the reliability and stability of the algorithm when dealing with complex problems in the real world. Lastly, the Gaussian distance decay function exhibits an increasing decay rate with distance, initially accelerating and then decelerating. This pattern is analogous to people's expectations in real-life decision-making processes regarding the selection of elderly care facilities. Therefore, it can simulate real decision-making states more realistically [30].

The calculation expression is as follows:

(4) $$\begin{array}{c} g\left(d_{ij}\right)=\frac{e^{-\left(1/2\right)}\times {\left(d_{ij}/d_{0}\right)}^{2}-e^{-\left(1/2\right)}}{1-e^{-\left(1/2\right)}},\;d_{ij}\leq d_{0}. \end{array}$$

#### 2.3.3. Spatial Autocorrelation

Spatial autocorrelation is commonly used to explore the presence of statistical correlation between data or variables in space or to investigate the potential mutual influence among several indicators. The research theory inherits from the first law of geography proposed by the Swiss geographer Waldo Tobler, i.e., everything is related to everything else, but near things are more related than distant things [31]. Spatial autocorrelation can reveal the distribution and pattern characteristics of data in space, such as exploring the aggregation and dispersion of data and identifying the hot and cold spots of data distribution. Therefore, it is often used to study the distribution of related indicators of public service facilities. In this study, the Global Moran's I and Getis–Ord General *G* indices were used to analyze the spatial distribution characteristics of the accessibility of ECSFs, and the Local Indicator of Spatial Autocorrelation (LISA) and Getis–Ord *G*_*i*_^∗^ tool were used to identify the areas of aggregation and dispersion of data.

##### 2.3.3.1. Global Spatial Autocorrelation

Global Moran's I reflects the overall spatial autocorrelation of the study area and is used to determine whether there is spatial autocorrelation in the research object as a whole. It is a spatial autocorrelation statistic for the entire study area. The Getis–Ord General *G* method is used to preliminarily determine the clustering type.

The Global Moran's I method of global spatial autocorrelation is given in the following equation:

(5) $$\begin{array}{c} I=\frac{n}{S_{0}}\times \frac{\sum_{i=1}^{n}\sum_{j=1}^{n}w_{ij}\left(y_{i}-\bar{y}\right)\left(y_{j}-\bar{y}\right)}{\sum_{i=1}^{n}{\left(y_{i}-\bar{y}\right)}^{2}},\;S_{0}=\overset{n}{\underset{i=1}{\sum }}\overset{n}{\underset{j=1}{\sum }}w_{ij}. \end{array}$$

The Getis–Ord General *G* method of global spatial autocorrelation is given in the following equation:

(6) $$\begin{array}{c} G=\frac{\sum_{i=1}^{n}\sum_{j=1}^{n}W_{i,j}x_{i}x_{j}}{\sum_{i=1}^{n}\sum_{j=1}^{n}x_{i}x_{j}},\;j\neq 1, \end{array}$$

where *y*_*i*(*j*)_ represents the accessibility calculation value obtained from residential area *i* (*j*) in this study; *y* represents the mean value of all research area data, which is the mean accessibility value of all residential areas in this study; *n* represents the number of residential areas; and *W* represents the spatial weight matrix [32].

##### 2.3.3.2. Local Spatial Autocorrelation

Global spatial autocorrelation statistics indicate the presence of clustering, while local spatial autocorrelation indicates the location and type of spatial association. To further study the distribution pattern of the spatial accessibility scores of ECSFs, this study used the LISA analysis method to identify local clusters of accessibility. Due to the heterogeneity of space, there may be different aggregation states in different geographical locations. LISA [33] is suitable for studying the heterogeneity characteristics of the clustering of ECSF accessibility. It calculates local indicators for each observation in the dataset and identifies four types of spatial association: (a) high–high (HH): locations with high attribute values surrounded by other locations with high attribute values. This indicates spatial clusters of similar values; (b) low–low (LL): locations with low attribute values surrounded by other locations with low attribute values. This also indicates spatial clusters of similar values; (c) high–low (HL): locations with high attribute values surrounded by other locations with low attribute values. This indicates spatial outliers or anomalies; and (d) low–high (LH): locations with low attribute values surrounded by other locations with high attribute values. This also indicates spatial outliers or anomalies.

The Getis–Ord *G*_*i*_^∗^ tool is suitable for hot spot analysis and can analyze the distribution of cold and hot spots of accessibility. The interpretation of the results is as follows: (a) positive *G*_*i*_^∗^ values: locations with positive *G*_*i*_^∗^ scores have high attribute values and are surrounded by other locations with high attribute values. This indicates statistically significant hot spots or areas of high clustering; (b) negative *G*_*i*_^∗^ values: locations with negative *G*_*i*_^∗^ scores have low attribute values and are surrounded by other locations with low attribute values. This indicates statistically significant cold spots or areas of low clustering; and (c) *Z*-scores close to zero: locations with *Z*-scores close to zero have attribute values similar to their neighbors, indicating no significant spatial clustering or dispersion.

The calculation expression is as follows.

The LISA method of local spatial autocorrelation is given in the following equation:

(7) $$\begin{array}{c} I_{i}=\frac{n\left(x_{i}-\bar{x}\right)\sum_{i=1}^{n}\sum_{j=1}^{n}w_{i,j}\left(x_{i}-\bar{x}\right)}{\sum_{i=1}^{n}{\left(x_{i}-\bar{x}\right)}^{2}}. \end{array}$$

The Getis–Ord *G*_*i*_^∗^ method of local spatial autocorrelation is given in the following equation:

(8) $$\begin{array}{c} G_{i}^{*}=\frac{\left.\sum_{j=1}^{n}W_{i,j}x_{j}-\overset{¯}{X}\sum_{j=1}^{n}w_{i,j}\right)}{S\sqrt[{2}]{\left(\left[n\sum_{j=1}^{n}w_{i}^{2}-{\left(\sum_{j=1}^{n}W_{i,j}\right)}^{2}\right]/n-1\right)}}, \end{array}$$

where *x*_*i*_ and *x*_*j*_ are attribute values for features *i* and *j*; *w*_*i*,*j*_ is the spatial weight between feature *i* and feature *j*; and *n* is the number of features in the dataset. When the *G*_*i*_^∗^ statistic is higher than the mathematical expectation and passes the hypothesis test, it is a hot spot; otherwise, it is a cold spot [32].

## 3. Results

### 3.1. Description

**Figure 4:**
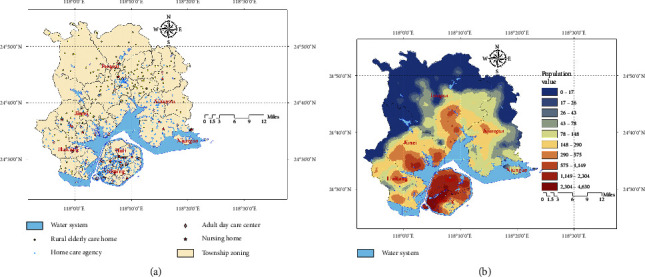
(a) The distribution of 660 ECSFs and (b) the distribution of the elderly population in Xiamen.

As of March 2023, there are 660 ECSFs in operation in Xiamen City, including 41 NHs and 44 ADCs, mainly located on Xiamen Island in the Huli and Siming districts. There are 441 HCAs, mainly distributed in population clusters on Xiamen Island and near the sea. There are also 134 REHs, mainly distributed in the Tong'an District. The specific distribution is shown in Figure 4(a). The Siming District has the highest concentration of elderly population, while the Xiang'an District has the lowest, and overall, the distribution shows radiation decay from the island to the outside, as shown in Figure 4(b).

### 3.2. Kernel Density Analysis

**Figure 5:**
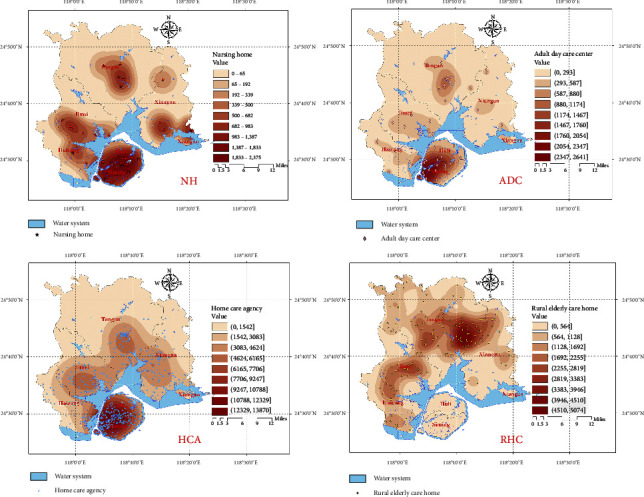
Result of kernel density analysis of ECSFs.

Kernel density analysis can show the spatial clustering of the distribution of ECSFs based on their quantity. The analysis results are shown in Figure 5, which reveals a serious clustering in the spatial distribution of various types of ECSFs in Xiamen City. NHs, which provide long-term care, are mainly concentrated in the Siming and Huli districts, with some clustering in the population clusters of Jimei and Tong'an, and lower clustering in other areas. ADCs, which mainly provide general care and companionship services, are mainly concentrated in the southwest of Xiamen Island. HCAs mainly provide on-site safety, daily living, and basic medical services to elderly people in surrounding communities, with main clusters in the Siming and Huli districts on Xiamen Island, but with the broadest coverage among all ECSFs. REHs mainly serve rural elderly people and provide comprehensive day care services such as daytime rest and leisure activities. They are mainly located outside Xiamen Island, with main clusters in the Tong'an and Jimei districts and few distributions on Xiamen Island.

According to the results of the kernel density analysis, the spatial distribution of existing ECSFs in Xiamen City is uneven, with most of them showing a clustering state centered on Xiamen Island. However, as kernel density analysis can only display the density quantity of various facilities in space, it cannot reflect the true relationship between the elderly population and the availability of elderly care resources. Therefore, it is necessary to use the Ga2SFCA method to further explore the accessibility of various ECSFs.

### 3.3. Accessibility Analysis

In order to fully consider the factor of aging population and further measure its true relationship with the availability of elderly care resources, this study used the Gaussian two-step mobile search method to evaluate the accessibility of ECSFs in Xiamen City.

**Figure 6:**
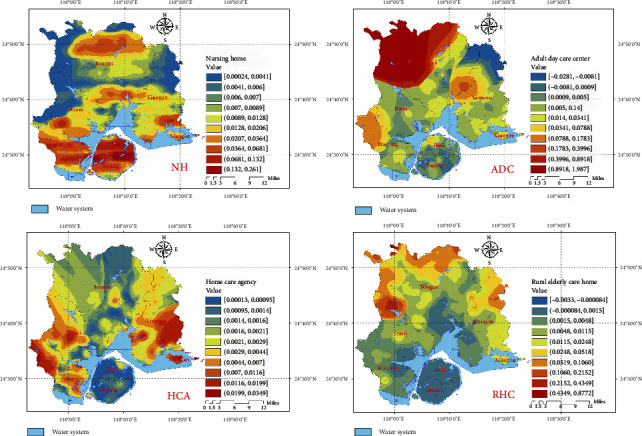
Accessibility analysis results of ECSFs.

Considering that Xiamen Island (Siming and Huli districts) and the surrounding areas (Haicang, Jimei, Tong'an, and Xiang'an districts) are separated by sea, this study set up path barriers around Xiamen Island. The detailed accessibility distribution results of ECSFs in Xiamen City were obtained and processed by Kriging interpolation, as shown in Figure 6. The high accessibility areas of NHs are mainly located in the midlatitude areas of Xiamen Island and Haicang District, while the low accessibility areas are mainly located in the northeast and northwest of Xiamen City. The high accessibility areas of ADCs are mainly located in the northwest, while the low accessibility areas are mainly located in the eastern areas. The low-value areas are mainly located on Xiamen Island, where HCAs are mainly clustered. The high-value areas of REHs are mainly located at the boundaries of Jimei, Tong'an, and Xiang'an districts, while the low-value areas are mainly located in the population clusters in the south of Xiamen Island and Tong'an District.

### 3.4. Global Spatial Autocorrelation

From the analysis of global spatial autocorrelation, it can be seen that the accessibility of the four types of ECSFs all show a significant clustering distribution state in space, with the REH showing the strongest positive correlation (Moran's *I* = 0.777853; observed General *G* = 0.005048) and the ADC showing the lowest positive correlation (Moran's *I* = 0.299089; observed General *G* = 0.009919). Details can be found in Table 3.

### 3.5. Local Spatial Autocorrelation Analysis

**Figure 7:**
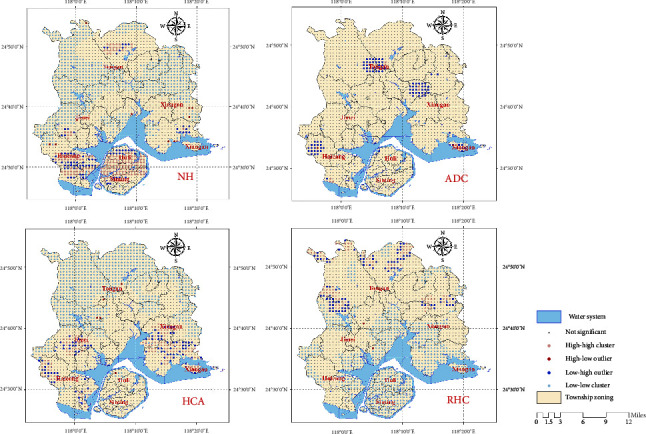
Clustering and outlier analysis results of various ECSFs.

**Figure 8:**
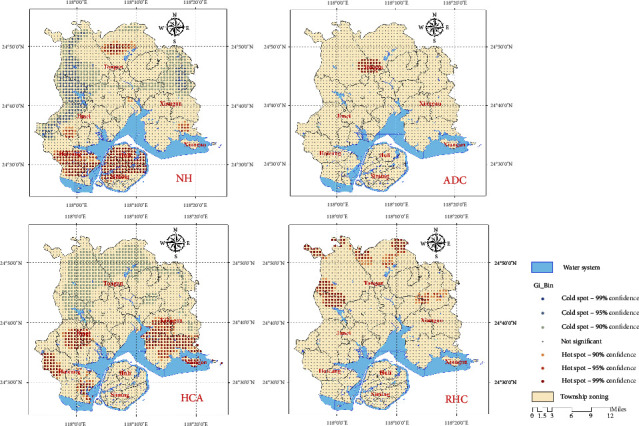
Analysis results of cold and hot spots of various ECSFs.

Based on the analysis of global spatial autocorrelation, this study used local spatial autocorrelation analysis to identify specific discrete and clustered areas in space. The results are as follows. The LISA map shows that the high–high clustering area of NHs in Xiamen City mainly appears in the central part of Xiamen Island and Haicang District, while the low–low clustering area is densely distributed in the midlatitude areas of Xiamen City. The high–high clustering area of elder care centers appears in Xiang'an and Tong'an districts, with large surrounding areas of low–high clustering. The high–high clustering area of HCAs mainly appears in the southern part of Haicang, Jimei, and Xiang'an districts, and there are widespread low–low clustering areas in the northern part of Xiamen City. The high–high clustering area of REHs mainly appears in the rural areas in the north of Xiamen City, and the low–low clustering area mainly appears in the southern part of Xiamen City (as shown in Figure 7). Hot spot analysis shows that the hot spot areas of NHs appear in Xiamen Island and Haicang District, and the east–west boundary of the midlatitude area in the city shows a cold spot distribution. The hot spot area of ADCs only appears in a small part of the central area of the Tong'an District. The hot spot areas of HCA mainly appear in the western part of Haicang District, Jimei District, and the central and eastern parts of Xiang'an District, while the cold spot areas mainly exist in the rural areas in the north of Xiamen City. The main hot spot area of REH appears in the boundary areas of the middle and northern parts of Xiamen City (Figure 8).

## 4. Discussion

As of March 2023, under the strong promotion of the country and the reasonable planning of the Xiamen municipal government, a series of achievements have been made in the construction of ECSFs in Xiamen. However, there are still gaps in the equity of elderly care resource utilization in achieving the goal of a 15-min living circle and the 2035 vision, and urgent efforts are needed to address this issue.

### 4.1. Distribution Patterns and Causes of ECSF in Xiamen City

Overall, the existing ECSFs in Xiamen demonstrate an imbalanced spatial distribution, with a higher concentration on Xiamen Island and fewer facilities in the surrounding areas. This pattern is characterized by the dominance of facilities on Xiamen Island and multiple core clusters in the areas outside the island. Specifically, NHs are primarily located in more prosperous areas, resulting in limited access to convenient long-term care for the elderly in most rural areas in the north of the city. This also poses challenges for family visits. The number of ADCs is insufficient, mainly concentrated in the Siming District, limiting the range of beneficiaries. HCAs primarily provide home-based security, daily living assistance, and basic medical care services to the elderly in surrounding communities. They are mainly concentrated in the Siming District and Huli District on Xiamen Island, with the widest coverage among all elderly care facilities. REHs primarily cater to rural elderly individuals, offering comprehensive daytime care services such as rest and recreational activities. They are mainly located outside Xiamen Island, with major clusters in the Tong'an District and Jimei District. They are less prevalent within Xiamen Island. Considering their similar functions to HCAs but with different target populations, their distribution is relatively reasonable.

There may be multiple factors contributing to this phenomenon. First, in terms of natural factors, the main topography of the Xiamen region consists of medium–low mountains, hills, plateaus, and coastal plains, generally exhibiting a northwest high to southeast low trend. The northwestern part of the area is characterized by mountains, hills, and hilly terrains, while the central–eastern part consists of plateaus and coastal plains [34]. The southeastern part comprises shallow tidal flats and artificially built-up plains. This leads to a preference for locating ECSFs in the southeastern part, making it difficult to establish clusters in the western regions. Second, in terms of historical factors, prior to the reform and opening-up, the main developed area of Xiamen was located on Xiamen Island. After the reform and opening-up, the urban area gradually expanded from the island toward the northern inland regions [35]. However, the distribution of ECSFs is deeply influenced by the urban development zones in Xiamen. The island area was developed earlier, with relatively complete supporting facilities, while the areas outside the island have a shorter development history, and both the coverage and construction density are in need of improvement. Third, in terms of economic development factors [36], Siming District and Huli District, located on Xiamen Island, are among the first batch of economic special zones in China and have a higher level of economic development. They are more likely to have a concentration of high-level public service facilities. The districts of Haicang, Jimei, Tong'an, and Xiang'an, located outside the island, have received focused planning in recent years, forming smaller scale clusters of ECSFs in their respective regional centers. However, in areas beyond these centers, the number of ECSFs is limited and their distribution is sparse. Lastly, while policy planning plays a guiding role in the rational allocation of ECSFs within the city, it can also influence the layout and equity of such facilities [36]. The supply of elderly care services comes from various sources, including the government, private capital, and social forces. These entities, driven by the pursuit of maximum benefits, make location choices for different types of ECSFs, leading to spatial disparities in the distribution of public service facilities [37]. For example, HCAs and REHs often involve government capital investment [38], resulting in more reasonable site selection that aligns well with their respective functions.

### 4.2. Existing Deficiencies and Potential Improvement Methods for the Accessibility of Various ECSFs

In terms of accessibility, the evaluation of NHs reveals that high-value areas are mainly located in Haicang District, Siming District, and Huli District, while large low-value areas are found in the peripheral rural areas in the northern part of Xiamen. Considering the government's recent efforts in developing the northern urban areas and the future direction of urban expansion toward the periphery, it is recommended that the government planning departments increase the construction of NHs in the peripheral areas. This would help increase the quantity and scale of facilities, contributing to a more rational allocation of public resources and greater equity in social services. If private enterprises refuse to establish NHs in rural areas, an alternative approach could be to develop corresponding NHs in suburban areas based on REHs. This would implement a “build first, optimize later” development approach, gradually achieving full coverage of NHs in rural areas and providing convenient long-term care services for rural residents.

Previous studies have indicated that ADCs, as the most important ECSFs at the community level in the context of community-based aging, are widely recognized by many older adults. A study conducted in Sichuan Province showed that over 60% of community-dwelling older adults expressed their willingness to choose the elderly care model provided by ADCs. However, the current situation in Xiamen shows that the construction of ADCs is not well planned, and there are even areas with negative values in terms of accessibility evaluation. This suggests that the policy planning departments should focus on the construction of ADCs in the future, expanding their quantity and coverage, especially in the northeastern part of Xiamen.

It is worth noting that the main construction areas of HCAs are located in Siming District and Huli District, but the accessibility evaluation shows that this area is a low-value area. This indicates that a significant portion of urban elderly residents are unable to conveniently access elderly care services due to the concentration of population and the small service radius of HCAs. Considering the limited geographical area of Xiamen Island, we suggest that unused houses and land in densely populated areas be converted into ECSFs. This approach would not only reduce the waste of community resources but also increase the density of relevant HCAs. It would make it easier for mobility-impaired elderly individuals to access the necessary care services in their living environment. In addition, converting or building new facilities can help alleviate the financial burden on the government's elderly care funds [39].

Indeed, in comparison, REHs have shown excellent accessibility evaluation results, with high-value areas primarily located in rural areas. In the future, it would be beneficial to expand the scale of existing REHs, improve their medical equipment and staffing, and expand their service coverage area. This would help overcome the unequal distribution of elderly care resources caused by geographical barriers. By expanding the reach of REHs, more elderly individuals in rural areas can access the necessary care services, ensuring a more equitable distribution of elderly care resources.

Furthermore, the research results indicate significant spatial heterogeneity in the accessibility of various types of ECSFs, characterized by distinct hot spots and cold spots. Since accessibility evaluation is based on specific road network distances, the establishment of a “15-min living circle” standard emphasizes walking as the primary mode of transportation for the elderly. The development of transportation infrastructure will have a significant impact on the accessibility of ADCs, HCAs, and REHs for older adults [40]. Therefore, enhancing the road network infrastructure in the areas outside Xiamen Island, particularly in the northwest and northeast rural regions, through measures such as increasing the number of road branches and opening up intersecting roads [41], would contribute to improving the convenience of elderly individuals in accessing care services and enhancing their overall well-being.

## 5. Limitations

Despite using multiple methods to comprehensively evaluate the accessibility of ECSFs in Xiamen City in this study, there are still some limitations: The elderly population in each region in this study was predicted and may deviate from the actual situation, the service range of elderly care facilities was set based on a large number of previous studies and government documents, and the research results match the actual situation in Xiamen City and can reveal accessibility issues, but there may be more appropriate numerical settings. In the future, we plan to use more real data to evaluate the spatial distribution equity of elderly care facilities nationwide.

## 6. Conclusions

This study evaluated the accessibility of elderly care services in Xiamen City based on the ECSFs and road traffic POI data obtained through web crawling technology. The results of this study have important guiding significance for managers and decision-makers to formulate relevant policies and achieve fair allocation of elderly care resources. The main discoveries are as follows: Xiamen City, as an economically developed city in Southeast China, has achieved a series of achievements in the allocation of elderly care resources and the construction of service facilities under the strong advocacy of the country and the reasonable planning of the local government. However, there are still problems of poor equity.

In order to improve the fairness of elderly resource allocation, optimize the accessibility of elderly care facilities, and enhance residents' well-being, this study proposes several recommendations. First, increasing the number of elderly care facilities should be accompanied by a focus on service quality. Second, efforts should be made to strengthen the road network infrastructure in areas outside Xiamen Island, particularly in the northwest and northeast rural regions. Third, it is suggested that government capital should be involved as a provider in the planning and construction of elderly care facilities. This participation of public capital can help reduce the service costs of elderly care facilities and mitigate their concentration, thus covering a wider range of elderly individuals and improving the overall level of elderly care services in Xiamen. Lastly, expanding the scale of existing rural happiness homes, improving their medical equipment and staffing, and expanding their service coverage area would overcome the unfair distribution of elderly care resources caused by geographical barriers.

## Figures and Tables

**Table 1:** Example of data acquisition results.

**Field name**	**Result**	**Paraphrase**
sOrgName	Huli Street (Haotou) Elderly Day Care Center	Institution name
sAddress	No. ^∗^, Hening 2nd Road (Building 1 and Building 4, Building 3, Jianfa Yibai Elderly Day Care Center)	Institution address
sTel	188∗∗∗∗5816	Contact information
iRunStatus	1	1: in operation; 0: suspended operation
sQualification	Public and private	Institutional nature
iOrgType	2	Institution type: 1 (NH); 2 (ADC); 3 (HCA); 4 (REH)
iBedCount	22	Number of beds
dCoveredArea	1000	Floor area (m^2^)
sLongitude	118.086046	Longitude
sLatitude	24.495159	Latitude

**Table 2:** The number of elderly population and the degree of aging in each district.

**District**	**Elderly population**	**Degree of aging (%)**
Siming	1,50,577	14.87
Huli	75,047	7.24
Haicang	46,362	7.96
Jimei	69,145	6.67
Tong'an	80,502	9.40
Xiang'an	62,946	10.90

*Note:* The data are sourced from the 7th National Population Census of China.

**Table 3:** Global autocorrelation analysis results.

**ECSF**	**Moran's I**	**Z-score**	***p*** ** value**	**Observed general G**	**Z-score**	***p*** ** value**
NH	0.34611	45.32629	< 0.00001	0.001312	50.4855	< 0.00001
ADC	0.299089	16.7353	< 0.00001	0.009919	12.25809	< 0.00001
HCA	0.362659	27.20153	< 0.00001	0.001932	48.85721	< 0.00001
REH	0.777853	39.9211	< 0.00001	0.005048	53.02876	< 0.00001

## Data Availability

The data for this study were obtained from publicly available government statistical yearbooks and public website POI data. The data used to support the findings of this study are available from the corresponding author upon request.
